# Temporal trends in fetal mortality at and beyond term and induction of labor in Germany 2005–2012: data from German routine perinatal monitoring

**DOI:** 10.1007/s00404-015-3795-x

**Published:** 2015-07-04

**Authors:** Christiane Schwarz, Rainhild Schäfers, Christine Loytved, Peter Heusser, Michael Abou-Dakn, Thomas König, Bettina Berger

**Affiliations:** Gerhard Kienle Lehrstuhl für Medizintheorie, Integrative und Anthroposophische Medizin, Institute for Integrative Medicine (IfIM), Universität Witten/Herdecke, Gemeinschaftskrankenhaus, Gerhard-Kienle-Weg 4, 58313 Herdecke, Germany; Midwifery Research and Education Unit, Department of Obstetrics, Gynaecology and Reproductive Medicine, Hannover Medical School, Carl-Neuberg-Str. 1, 30625 Hannover, Germany; Department of Applied Health Sciences, University of Applied Sciences, Universitätsstraße 105, 44789 Bochum, Germany; School of Health Professions, Institute of Midwifery, Zurich University of Applied Sciences, Winterthur, Switzerland; Studiengang Hebammenkunde, Evangelische Hochschule Berlin, Teltower Damm 118-122, 14167 Berlin, Germany; AQUA-Institut für angewandte Qualitätsförderung und Forschung im Gesundheitswesen GmbH, Maschmühlenweg 8-10, 37073 Göttingen, Germany

**Keywords:** Fetal mortality, Perinatal data, Postmaturity, Post-dates, Induction of labor

## Abstract

**Purpose:**

While a variety of factors may play a role in fetal and neonatal deaths, postmaturity as a cause of stillbirth remains a topic of debate. It still is unclear, whether induction of labor at a particular gestational age may prevent fetal deaths.

**Methods:**

A multidisciplinary working group was granted access to the most recent set of relevant German routine perinatal data, comprising all 5,291,011 hospital births from 2005 to 2012. We analyzed correlations in rates of induction of labor (IOL), perinatal mortality (in particular stillbirths) at different gestational ages, and fetal morbidity. Correlations were tested with Pearson’s product-moment analysis (*α* = 5 %). All computations were performed with SPSS version 22.

**Results:**

Induction rates rose significantly from 16.5 to 21.9 % (*r* = 0.98; *p* < 0.001). There were no significant changes in stillbirth rates (0.28–0.35 per 100 births; *r* = 0.045; *p* = 0.806). Stillbirth rates 2009–2012 remained stable in all gestational age groups irrespective of induction. Fetal morbidity (one or more ICD-10 codes) rose significantly during 2005–2012. This was true for both children with (from 33 to 37 %, *r* = 0.784, *p* < 0.001) and without (from 25 to 31 %, (*r* = 0.920, *p* < 0.001) IOL.

**Conclusions:**

An increase in IOL at term is not associated with a decline in perinatal mortality. Perinatal morbidity increased with and without indiction of labor.

## Introduction

While prematurity and congenital anomalies remain the leading cause of perinatal mortality and morbidity, there are also fetal deaths of mature, apparently normally formed babies. There has been a debate about factors causing late stillbirths in this population including postmaturity. Induction of labor (IOL) at a specific gestational age may prevent some of those deaths, with or without influencing the rate of Cesarean sections [1–9]. Current studies suggest a multifactorial explanation for fetal deaths, such as maternal risk factors like primiparity, hypertension and diabetes, advanced age, obesity, or smoking. As far as fetal risks are concerned, fetuses of relatively low and high weight seem to be at higher risk than average-sized babies, pointing to growth retardation as well as diabetic complications in pregnancy as relevant risk factors [10, 11]. As Warland et al. [12] discuss in their recent publication, combinations of any of those risk factors with an acute stressor like venocaval compression may then lead to fetal demise. Also, a cluster of genetic predispositions correlated with ethnicity may influence the risk of stillbirth [13, 14]. Infections are another cause of fetal death and severe neonatal morbidity [15]. The role of a previous Cesarean section needs to be further evaluated [16]. Even an environmental influence of seasonal temperature changes on stillbirth rates has been discussed [17].

With all these approaches aiming to analyze different causes of fetal death, the role of postmaturity as a cause of perinatal mortality is another open question. In fact, there is still debate about the ideal gestational age for delivery and how to statistically measure stillbirth rates [18–24]. The “fetus-at-risk” model calculates the risk of fetal death per ongoing pregnancy rather than per 1000 births in that gestational week [18, 19, 21, 22, 25, 26]. The incidence of adverse perinatal outcomes due to prematurity drops with advancing gestational age, while the risk of fetal death due to postmaturity seems to rise as pregnancy progresses once term is reached. To reliably determine the risk of perinatal death—in particular, stillbirth—due to postmaturity, or to calculate an individual risk for a particular fetus seems impossible on these grounds. Recent clinical guidelines suggest to advise or offer induction of labor once the gestational age of 41 + 0 is reached [27–29]. However, the insecurity to exactly determine the gestational age in an individual pregnancy, the variety of factors leading to fetal demise, and the ongoing debate on the correct calculation of fetal risk makes a clear recommendation for an individual pregnant woman difficult. Also, there may be harmful impacts on neonatal morbidity with IOL at term that needs to be addressed [30]. In the light of these open questions, it seems reasonable to take a look at the development of IOL rates and rates of perinatal mortality and morbidity on a population level in recent data to find out whether or not there may be a correlation between rates of labor induction and fetal and neonatal health outcomes.

## Method

A multidisciplinary working group based at Universität Witten/Herdecke was granted access to the most recent German routine perinatal data with the Federal Joint Committee, (“Gemeinsamer Bundesausschuss,” G-BA) in 2013. According to University of Witten Ethics Committee and German Federal Joint Committee, formal ethical approval was not necessary, as retrospective perinatal data were collected and displayed under German data protection laws. Data collection covered all hospital births (approximately 98.5 % of all births) in Germany. Until 2008, routine perinatal data were processed and published by the National Institute for Quality and Patient Safety (“Institut für Qualität und Patientensicherheit,” BQS); since 2009, the Institute for Applied Quality Improvement and Research in Health Care (AQUA) is responsible for data collection and publication within the nationwide cross-sectoral healthcare quality assurance. The perinatal dataset contains 190 items regarding pregnancy and birth process and outcome indicators. Due to data protection laws, linking of individual datasets from the perinatal and the neonatal registers is not possible. Between 2005 and 2012, perinatal datasets of 5,291,011 births were recorded. Data encompassed

1. IOL frequency and rate,
2. Gestational age of IOL,
3. “post-term” as the reason for induction of labor,
4. Fetal death rate (whole sample and sample >36 + 6 gestation excluding multiple pregnancies and fetuses with known structural abnormalities) with and without IOL,
5. Fetal death rate at particular gestational ages (whole sample and sample >36 + 6 gestation excluding multiple pregnancies and fetuses with known structural abnormalities),
6. Neonatal morbidity (all diagnoses and hypoxia/breathing irregularities) in relation with gestational age and IOL, and
7. Maternal morbidity in relation to IOL and maternal age.

The data were read into IBM SPSS version 22, and Pearson product-moment correlation coefficient was considered significant below the level of *α* = 5 %.

## Results

1. The induction of labor (IOL) rates in all 5,291,011 pregnancies changed between 2005 and 2012, showing a steady rise from 16.5 % (*n* = 109,709) in 2005 to 21.9 % (*n* = 145,265) in 2012. This rise was almost perfectly linear and highly significant (*r* = 0.983, *p* < 0.001) (Fig. 1).

**Fig. 1:**
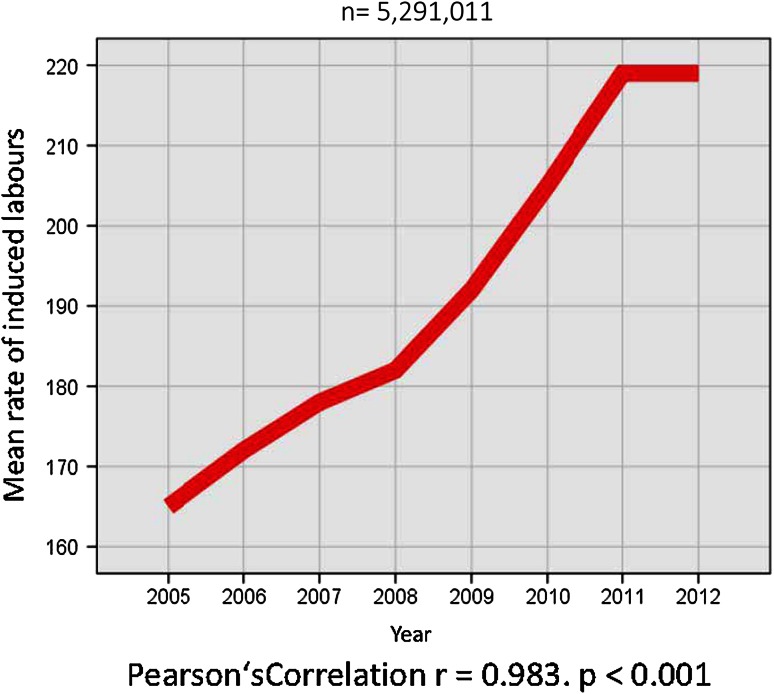
Rate of IOL 2005–2012 per 1000 deliveries
2. The mean gestational age at induction of labor changed significantly between 2005 (41 + 0 gestation) and 2012 (39 + 5 gestation) (*r* = 0.920, *p* < 0.001). This change did not develop evenly over time, but with a steep drop between 2008 and 2009. The re-calculation for 2009–2012 showed no change in mean gestational age.
3. Postmaturity as the given reason for IOL showed a similar pattern as the mean gestational age, with an even rate between 2005 and 2008 (mean 75.5 %), and then again an even rate between 2009 and 2012 on a very different level (mean 54.4 %). Pearson correlation over time is significant (*r* = 0.646, *p* = 0.007).
4. Fetal death rate for all births (*n* = 17,455 versus 5,291,011 live births) remained stable between 2005 (0.331 %) and 2012 (0.334 %) with no significant changes or even trends (*r* = 0.045, *p* = 0.806). There was a marked decrease in stillbirth rates in the third quarter (July–September: mean 0.3026, SD 0.13) of each year, compared to the months January–June and October–Dec (mean 0.34, SD 0.13), while at the same time birth rates during these months were peaking (Fig. 2). *T* test shows that this difference is highly significant (*p* < 0.001).

**Fig. 2:**
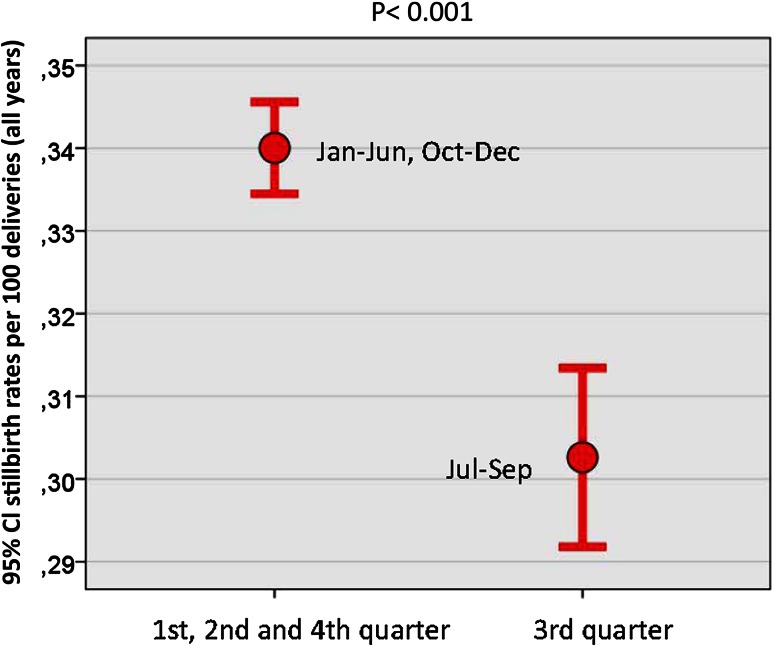
Seasonal changes in stillbirth rates 2005–2012
5. When stillbirth rates 2005–2012 were calculated for singleton pregnancies later than >36 + 0 gestation (*n* = 5,622,515), rates showed an increase from 0.19 % in 2005 to 0.29 % in 2012 (*p* < 0.001). This was true for both infants with induced (*n* = 5134 stillbirths versus 1,100,096 live births) and non-induced (*n* = 5134 stillbirths versus 4,505,543 live births) labors. Mean fetal death rates for induced labors were 0.77 % versus 0.11 % for non-induced pregnancies. Early neonatal death rates showed a similar increase in both groups (Table 1; Fig. 3).

**Table 1:** Perinatal mortality with and without induction of labor, Germany, 2005–2012

Count	In-hospital mortality × induction of labor × year cross-tabulation				
	Induction of labor				Total
Year	No IOL	Rate (%)	IOL	Rate (%)	
2005					
In-hospital mortality					
Live	516,902		103,186		620,088
Fetal death	483	0.09	671	0.65	1154
Neonatal death	180	0.03	44	0.04	224
Total	517,565		103,901		621,466
2006					
In-hospital mortality					
Live	503,741		105,991		609,732
Fetal death	422	0.08	625	0.59	1047
Neonatal death	181	0.04	57	0.05	238
Total	504,344		106,673		611,017
2007					
In-hospital mortality					
Live	508,125		111,745		619,870
Fetal death	366	0.07	686	0.61	1052
Neonatal death	198	0.04	44	0.04	242
Total	508,689		112,475		621,164
2008					
In-hospital mortality					
Live	509,270		114,818		624,088
Fetal death	499	0.10	802	0.69	1301
Neonatal death	242	0.05	50	0.04	292
Total	510,011		115,670		625,681
2009					
In-hospital mortality					
Live	498,333		118,916		617,249
Fetal death	609	0.12	1137	0.95	1746
Neonatal death	403	0.08	96	0.08	499
Total	499,345		120,149		619,494
2010					
In-hospital mortality					
Live	501,435		130,372		631,807
Fetal death	703	0.14	1120	0.85	1823
Neonatal death	387	0.08	95	0.07	482
Total	502,525		131,587		634,112
2011					
In-hospital mortality					
Live	480,996		136,249		617,245
Fetal death	656	0.14	1137	0.83	1793
Neonatal death	362	0.08	69	0.05	431
Total	482,014		137,455		619,469
2012					
In-hospital mortality					
Live	490,151		138,666		628,817
Fetal death	701	0.14	1130	0.81	1831
Neonatal death	327	0.07	92	0.07	419
Total	491,179		139,888		631,067
Total					
In-hospital mortality					
Live	4,505,543		1,100,096		5,605,639
Fetal death	5134	0.11	8499	0.77	13,633
Neonatal death	2609	0.06	634	0.06	3243
Total	4,513,286		1,109,229		5,622,515

**Fig. 3:**
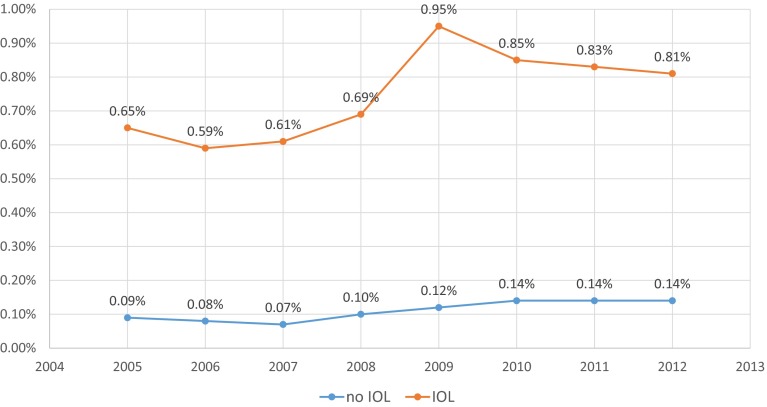
Fetal mortality in induced and non-induced singleton pregnancies >36 + 6 gestation per 100 deliveries, Germany 2005–12
6. Neonatal morbidity between 2005 and 2012 was defined as every infant diagnosed with ICD10 code during this time interval. Obviously, morbidity rates (ICD-10 codes, without/with IOL) in term neonates differ according to maturity, with the highest rates in early term infants before 38 + 0 gestation (7.12 %/10.33 %) and later than 42 completed weeks (10.97 %/12.65 %). The lowest rates are 39 + 0–39 + 6 gestation (4.77 %/7.02 %). When we restricted the analysis for term neonates to hypoxia/respiratory distress (ICD-10 P 20–22), rates 2005–2012 decreased for both induced (1.02–0.48 %) and non-induced (0.74–0.38 %) infants, but at the same time other diagnoses increased in both groups, adding up to a net increase for total morbidity in term neonates over the study period (7.54–9.09 % for IOL and 5.26 to 7.16 % without induction, Pearson’s correlation r = 0.976 (*p* < 0.01)) (Table 2; Fig. 4).

**Table 2:** Morbidity in term neonates, Germany 2005–2012, for infants with and without induction of labor

Year	Diagnosis	No IOL	Rate	IOL	Rate (%)
2005	None	490,303	95	96,063	92.46
	P20–22	3852	0.74 %	1059	1.02
	Only other	23,410	4.52 %	6779	6.52
	Total morb		5.26 %		7.54
	Total	517,565		103,901	
2006	None	476,566	94.49 %	98,546	92.38
	P20–22	3755	0.74 %	1063	1.00
	Only other	24,023	4.76 %	7064	6.62
	Total	504,344	5.50 %	106,673	7.62
2007	None	479,891	94.34 %	103,269	91.82
	P20–22	2586	0.51 %	822	0.73
	Only other	26,212	5.15 %	8384	7.45
	Total morb		5.66 %		8.18
	Total	508,689		112,475	
2008	None	480,538	94.22 %	106,309	91.91
	P20–22	1954	0.38 %	628	0.54
	Only other	27,519	5.40 %	8733	7.55
	Total morb		5.78 %		8.09
	Total	510,011		115,670	
2009	None	466,239	93.37 %	109,767	91.36
	P20–22	2187	0.44 %	639	0.53
	Only other	30,919	6.19 %	9743	8.11
	Total morb				
	TOTAL	499,345	6.63 %	120,149	8.64
2010	None	468,549	93.24 %	119,865	91.09
	P20–22	2129	0.42 %	691	0.53
	Only other	31,847	6.34 %	11,031	8.38
	Total morb		6.76 %		8.91
	Total	502,525		131,587	
2011	None	447,873	92.92 %	125,064	90.99
	P20–22	1976	0.41 %	696	0.50
	Only other	32,165	6.67 %	11,695	8.51
	Total morb		7.08 %		9.01
	Total	482,014		137,455	
2012	None	456,011	92.84 %	127,178	90.91
	P20–22	1890	0.38 %	671	0.48
	Only other	33,278	6.78 %	12,039	8.61
	Total morb		7.16 %		9.09
	Total	491,179		139,888	

P20–22 = hypoxia/respiratory distress, only other = all diagnoses excluding P20–22

**Fig. 4:**
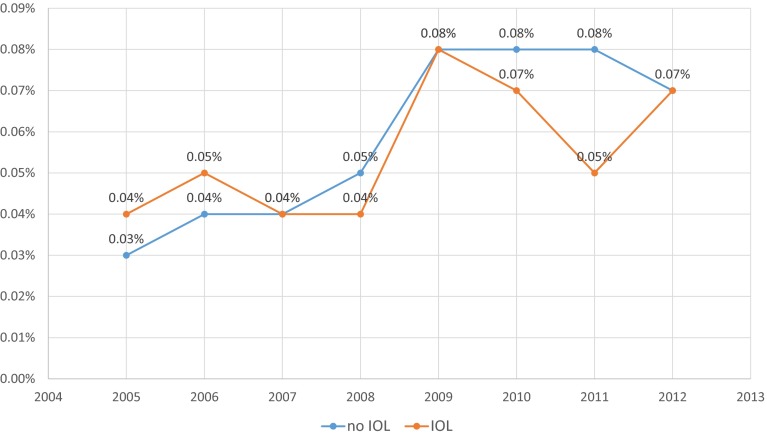
Early neonatal mortality in induced and non-induced singleton pregnancies >36 + 6 gestation, Germany 2005–2012
7. Maternal morbidity rates were calculated using any documented maternal complication. These were perineal trauma, hemorrhage, peripartum hysterectomy, eclampsia, sepsis, and other. Morbidity rates in relation to gestational age at delivery as well as in relation to IOL showed an inexplicable, steep drop between 2007 and 2008. Analysis was then restricted to 2008–2012. For women with no IOL rates remained stable over the period, with approximately half of the study population experiencing no complications. For women who were induced, the rate of complications increased steadily from 51.24 to 55.97 %. This finding was significant (*p* < 0.001). Maternal age was not documented before 2006. Mean maternal age increased during the period 2006–2012 from 29.45 to 30.22 years. This rise correlated with the increase in fetal death rate and neonatal mortality and morbidity (*r* = 0.882, *p* < 0.01) (Table 3; Fig. 5).

**Table 3:** Maternal morbidity × all complications × with and without IOL, Germany, 2005–2012

Year		No IOL	Rate (%)	IOL	Rate (%)	Mean maternal age
2005						
No comp		262,141	50.65	50,660	48.76	No data available
Any comp		255,424	49.35	53,241	51.24	
Total		517,565		103,901		
2006						
No comp		254,088	50.38	51,085	47.89	29.45
Any comp		250,256	49.62	55,588	52.11	
Total		504,344		106,673		
2007						
No comp		263,718	51.84	54,648	48.59	29.88
Any comp		244,971	48.16	57,827	51.41	
Total		508,689		112,475		
2008						
No comp		264,954	51.95	55,086	47.62	29.96
Any comp		245,057	48.05	60,584	52.38	
Total		510,011		115,670		
2009						
No comp		259,015	51.87	56,319	46.87	30.05
Any comp		240,330	48.13	63,830	53.13	
Total		499,345		120,149		
2010						
No comp		258,088	51.36	60,582	46.04	30.14
Any comp		244,437	48.64	71,005	53.96	
Total		502,525		131,587		
2011						
No comp		245,318	50.89	61,849	45.00	30.21
Any comp		236,696	49.11	75,606	55.00	
Total		482,014		137,455		
2,012						
No comp		245,727	50.03	61,599	44.03	30.22
Any comp		245,452	49.97	78,289	55.97	
Total		491,179		139,888

**Fig. 5:**
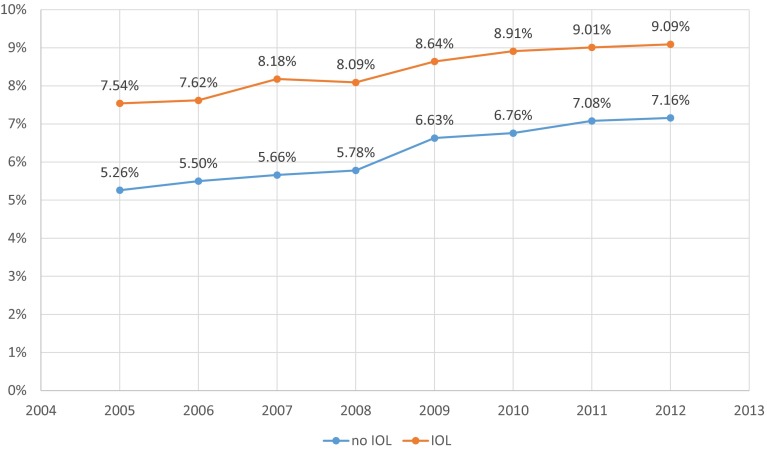
Total morbidity in singleton neonates >36 + 6 gestation, Germany 2005–2012

## Discussion

Our results do not show a correlation between fetal death, gestational age and a possible benefit of induction of labor at term. Some patterns of fetal deaths, like seasonal changes in stillbirth rates, remain inexplicable. Within all the post-term discussion, there is still little mention of other causes (like maternal age, infections, growth retardation or macrosomia, maternal sleeping positions, and other) being responsible for the majority of stillbirths [11, 12, 14]. Study results on perinatal/fetal mortality informing clinical guidelines are often based on retrospective secondary data that have not been adjusted to those factors. Due to these study designs, it has—in spite of extensive research—to date been impossible to determine definite causal variables for most fetal deaths [10, 11, 20, 31–33]. The rareness of the event of a fetal death adds to the complexity of interpreting study results—one case more or less makes for a huge difference in death rates. In the absence of robust knowledge on causes for fetal deaths in normal pregnancies, many clinical guidelines offer or recommend IOL at or after a certain gestational length, relating to the interpretation of secondary statistical data [28, 34–36]. On the assumption that stillbirth rates increase after 41 + 0 gestational age, IOL appears to be the solution to prevent those fetal deaths. If that was the case, stillbirth rates should decline when IOL rates at or after that date increase.

We applied for the complete dataset of all 5,291,011 births with no exclusion criteria, because we wanted to be able to check for “surprises” (like the change in definitions/diagnostic to determine gestational age). To ensure comparability with other published data, a decision was made not to adjust for known variables related to stillbirths, like socio-economic status, drug consumption, and diagnosis of gestational diabetes (universal screening for gestational diabetes has only become part of routine German antenatal care in 2012). While analyzing the data, some of the above outcome parameters showed implausibility that could not be explained by experts. There were marked differences between the cluster years 2005–2008 and 2009–2012, which corresponded with the change in both data processing institutes BQS and AQUA in Germany. This sharp drop from one quarter to the other may be caused by a methodological artifact. Due to this finding, the working group consented in re-calculating the data for the years 2009–2012 (*n* = 2,626,997), where appropriate. We calculated sub-samples (singleton term pregnancies), where appropriate. We cannot explain a steep rise in fetal death rate after 2008 due to possible artifacts with data processing by two different institutions. When we looked for an explanation for increasing morbidity rates from 2009 to 2012, we included maternal age and found that we found that mean maternal age increased from 29.45 to 30.22 years. This finding correlates significantly with the increase in neonatal morbidity (*r* = 0.882, *p* < 0.01). Morbidity in the dataset contains all ICD-10 codes between P00 and P96 (problems originating in the perinatal period) documented by the hospitals. These are mainly infections, respiratory problems, or hypoxia. We decided not to focus on meconium aspiration (as a typical scenario associated with postmaturity) only, as we did not want to miss other potential adverse effects of induction or watchful waiting that may outweigh benefits. Still, we calculated morbidity for infants with and without IOL and for ICD-10 codes P20–22 (hypoxia/respiratory distress). While morbidity in term infants increased in both induced and not induced pregnancies, maternal complications increased in induced women only. Data do not prove a causal relationship between maternal and neonatal morbidity and IOL (was IOL initiated because of maternal complications, followed by neonatal morbidity, or does maternal and neonatal morbidity increase due to IOL). Induction may occasionally have been offered to older women over the study period, although the German guidelines do not suggest a particular maternal age for earlier induction. A causal link between IOL and possible changes in morbidity or mortality for infants or mothers cannot be proven with our study design. The increases in neonatal and maternal morbidity could be attributed to the increase in maternal age. Maternal age increased slightly during this period. Still, other factors cannot be determined, as the diagnosis and definition for complications like gestational diabetes changed during the study period, leading to incomparable results. As more than one indication for IOL can be documented in the data, reasons like “maternal request” are not included in the documentation, and “postmaturity” may be documented for term pregnancies between 40 + 0 and 41 + 3 gestational weeks, it is difficult to link indications to outcomes. We argue that the increase in IOL has not been accompanied by a decline in morbidity or mortality for neither women nor babies. So, the recommendation to induce more liberally cannot be supported by our data. Our results do not show a correlation between fetal death, gestational age and induction of labor at term and therefor no benefit regarding the reduction of the fetal death rate.

As the overall incidence of fetal death has been historically low in developed nations over the past decades, the current rates may even reflect the limit of avoidable cases. Very unlikely will there ever be a zero rate for stillbirths. We need to take into consideration possible factors for the significant rise of maternal/fetal morbidity when we intervene in a healthy pregnancy hoping to maybe prevent a stillbirth, and we need to counsel women on the grounds of solid evidence.

## Conclusions

Our data show that IOL at and shortly beyond term is not correlated with a decline in perinatal mortality, in particular not with fetal deaths. We noticed a rise in both neonatal and maternal morbidity within the time period 2005–2012 or, where appropriate due to data quality, during 2009–2012. Our findings indicate that in the absence of solid understanding of all risk factors for stillbirth, an intervention which may lack some proof to be more beneficial to both mothers and children than to “watch and wait,” like routine liberal use of IOL in healthy term women, should be outweighed carefully for potential harm and benefit. Our data analysis does not support early term IOL in healthy women. There is a lack of data on women’s views and their experiences with induction of labor. Evaluation of stillbirth cases for underlying causes and the effectiveness of different pathways for expectant management need to be further explored.
